# The Maryland Dental Bill

**Published:** 1884-02

**Authors:** 


THE MARYLAND DENTAL BILL.—A Dental Bill to Regulate the Practice of Dentistry in the State of Maryland has passed the House of Delegates (February 15th, 1884), and been read in the Senate. As this bill may yet be somewhat modified at its second reading in the Senate, we will defer its publication in order to give the readers of the JOURNAL a correct copy of this Act in the March number. As the Bill now stands it is a compromise between the two bills presented respectively by the State Society, and that styling itself the Dental Legislative Society, but the latter's (like Othello's) occupation is gone, as all of the vital points of their bill have been omitted in the one now on its passage. The Governor of the State appoints the Board of Examiners without recommendations from any Society: the said Board has no power over any diplomas granted by dental institutions of this State: there is no Examiner's pay provided for by the Bill, and in fact all the principal features of both bills have been omitted and a bill made up from both by the Judiciary Committe of the House of Delegates. So far as we are concerned, although it is not such a bill as we should desire, yet it is better than no bill, and may do some good. A much better bill was presented by the State Association, and there is no doubt whatever that it would have passed both House and Senate had it not been for the opposition of the society which was formed to antagonize the action of the State Association. We would prefer a Board of Examiners consisting of dental graduates, as we hold to the opinion that such graduates are much better qualified to act in that capacity than others who have not passed through such a curriculum, with but very few exceptions. The anomaly of subjecting educated men to an examination by uneducated ones, who may owe their appointment as a Board of Examiners to political influence or favor, is very apparent. On the whole, however, any discrepancies in this bill can be justly laid upon the heads of those who opposed the action of the State Dental Association in this matter, and we have the consolation of feeling that the principal objections existing against the bill presented by the so-called Dental Legislative Society, have been omitted in the bill likely to become a law within the next few days.

